# Phytochemical Composition, Antioxidant and Xanthine Oxidase Inhibitory Activities of *Amaranthus cruentus* L. and *Amaranthus hybridus* L. Extracts

**DOI:** 10.3390/ph5060613

**Published:** 2012-06-15

**Authors:** Fernand W. Nana, Adama Hilou, Jeanne F. Millogo, Odile G. Nacoulma

**Affiliations:** 1 Laboratoire de Biochimie et de Chimie Appliquées, UFR/SVT, Université de Ouagadougou, 03 BP 7021 Ouaga 03, Burkina Faso; Email: fernandnana@gmail.com (F.W.N) ; hiloudio@gmail.com (A.H.); odilenacoulma@yahoo.com (O.G.N.); 2 Laboratoire de Biologie et d’Ecologie Végétales, U.F.R./S.V.T., Université de Ouagadougou, 03 BP 7021 Ouaga 03, Burkina Faso; Email: fremiotdc@yahoo.fr (J.F.M.)

**Keywords:** nutraceutical, phenolics, betalain, antioxidant, *amaranthus*

## Abstract

This paper describes a preliminary assessment of the nutraceutical value of *Amaranthus cruentus (A. cruentus)* and *Amaranthus hybridus (A. hybridus)*, two food plant species found in Burkina Faso. Hydroacetonic (HAE), methanolic (ME), and aqueous extracts (AE) from the aerial parts were screened for *in vitro* antioxidant and xanthine oxidase inhibitory activities. Phytochemical analyses revealed the presence of polyphenols, tannins, flavonoids, steroids, terpenoids, saponins and betalains. Hydroacetonic extracts have shown the most diversity for secondary metabolites. The TLC analyses of flavonoids from HAE extracts showed the presence of rutin and other unidentified compounds. The phenolic compound contents of the HAE, ME and AE extracts were determined using the Folin–Ciocalteu method and ranged from 7.55 to 10.18 mg Gallic acid equivalent GAE/100 mg. Tannins, flavonoids, and flavonols ranged from 2.83 to 10.17 mg tannic acid equivalent (TAE)/100 mg, 0.37 to 7.06 mg quercetin equivalent (QE) /100 mg, and 0.09 to 1.31 mg QE/100 mg, respectively. The betacyanin contents were 40.42 and 6.35 mg Amaranthin Equivalent/100 g aerial parts (dry weight) in *A. cruentus* and *A. hybridus*, respectively. Free-radical scavenging activity expressed as IC_50 _(DPPH method) and iron reducing power (FRAP method) ranged from 56 to 423 µg/mL and from 2.26 to 2.56 mmol AAE/g, respectively. Xanthine oxidase inhibitory activities of extracts of *A. cruentus* and *A. hybridus* were 3.18% and 38.22%, respectively. The *A. hybridus* extract showed the best antioxidant and xanthine oxidase inhibition activities. The results indicated that the phytochemical contents of the two species justify their traditional uses as nutraceutical food plants.

## 1. Introduction

In developing countries such as Burkina Faso, a large part of the population has the habit of consuming traditional food plants. Recent phytochemical analyses have shown that most plants consumed contain several health protecting nutrients (nutraceuticals). The popularity of nutraceuticals and traditional medicines are soaring as consumers take a keener interest in health and nutrition. As markets for these products develop, tighter regulations are being introduced to ensure quality, efficacy and safety. Ethnobotanical and traditional food plant investigations in the central region of Burkina Faso have shown that approximately thirty Caryophyllales species are widely and frequently used in traditional medicine to treat various kinds of diseases (e.g., malaria, fever, pain, hepatic disorders, nervous system issues, cancers, and cardiovascular diseases) and are also consumed as food plants [1]. Phytochemical screening (qualitative and quantitative) by laboratory standard means should allow for the validation of their nutraceutical potential. The present study concerns *Amaranthus cruentus* L. and *Amaranthus hybridus* L., two species from the Amaranthaceae family (Caryophyllales).

*Amaranthus* spp*.* were of great importance in pre-Colombian American people’s diets [2,3]; in particular, *A. cruentus* and *A. hybridus* have a high nutritional value [4,5,6,7,8,9,10]. The consumption of *A. cruentus* products is advised for patients with celiac disease and, therefore, also for diabetic persons [8]. *A. hybridus* has been used traditionally for the treatment of liver infections and knee pain and for its laxative, diuretic, and cicatrisation properties [11]; the products are used particularly for stomach aches, diarrhoea, and dysentery. *A. hybridus* leaves are used as a vegetable [12], and sauces prepared from this plant are recommended for convalescent patients [1]. These two species are reputed to promote health, and both have a long shelf life. 

A healthy organism has natural antioxidant tools [13], including antioxidant enzymes, such as glutathione peroxidase, catalase, and superoxide dismutase, and antioxidant compounds, such as glutathione, tocopherol and ascorbic acid [14]. When the natural physiological antioxidant capacity is exceeded, the natural antioxidant mechanisms may be reinforced by the consumption of plants rich in antioxidant compounds, such as vitamins (e.g., ascorbic acid, tocopherols, and carotenoids), polyphenols [15] and betalains [1]. Research has shown a positive correlation between natural antioxidant compound consumption and a decrease in cardiovascular diseases and cancers. To assess their nutraceutical properties, a study was conducted to investigate the phytochemicals and antioxidant capacities of *A. cruentus* and *A. hybridus* extracts. 

## 2. Results and Discussion

### 2.1. Phytochemical Results

Fresh *A. cruentus* and *A. hybridus* moisture rates and dried plant material extraction yields from extractions using aqueous acetone (*i.e*., hydroacetonic) , methanolic or water as a solvent are presented in Table 1. *A. cruentus* and *A. hybridus* contain remarkably high amounts of moisture, at 80.86 ± 1.18% and 83.45 ± 0.99%, respectively. Odhav *et al.* [6] and Aletor and Adeogun [7] reported 83% moisture for *A. hybridus* and 81.3% for *A. cruentus*. The two species possess a C4 photosynthetic system [16], which is known to be adapted for tropical regions. The amount of extractable components ranged from 18.55% in *A. cruentus* (with AE) to 7.43% in *A. hybridus* (with ME). *A. cruentus* exhibited the best yields for the three types of extractions. In this study, the water extract investigation was important, as it represents the most used traditional preparation for these species.

**Table 1:** Moisture and extract yields (from dry plant materials) of studied vegetables.

Species	Moisture (%)	Yields (%)		
		HAE	ME	AE
*A. cruentus*	80.86 ± 1.18	10.50	8.99	18.55
*A. hybridus*	83.45 ± 0.99	8.59	7.43	12.23

HAE: hydroacetonic extract ▬ ME: methanolic extract ▬ AE: aqueous extract.

Phytochemical analyses revealed the presence of polyphenols, tannins, flavonoids, steroids (including cardenolids), terpenoids (i.e., iridoïds, triterpenes and carotenoids), saponins and betalains (Table 2). 

**Table 2:** Phytochemical composition of *A. cruentus* and *A. hybridus*.

	*A. cruentus*					*A. hybridus*			
	HAE	ME		AE		HAE	ME		AE
- Polyphenols and tannins	+	+		+		+	+		+
- Flavonoids	+	+		-		+	+		-
- Anthracenosides	-	-		-		-	-		-
- Coumarins	-	-		-		-	-		-
- Steroids and triterpenes	+	+		+		+	+		+
- Iridoïds	+	+		+		+	+		+
- Cardenolids	+	-		-		+	-		-
- Carotenoids	+	+		-		+	+		-
- Sapononins	-	-		+		-	-		+
- Alkaloids	-	-		-		-	-		-
	WAE		AE			WAE		AE	
- Betalains	+		-			+		-	

(-): non identified; (+): identified; HAE: hydroacetonic extract; ME: methanolic extract; AE: aqueous extract; WAE: water ascorbate extract.

**Figure 1:**
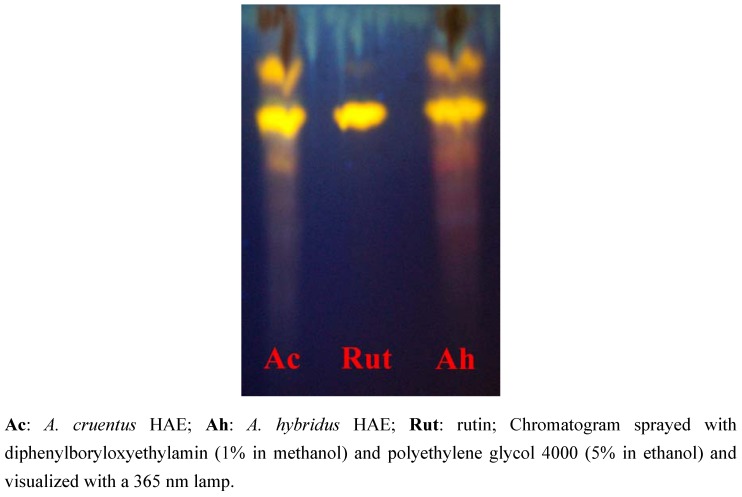
Thin-layer chromatography results.

The thin layer chromatography analyses of flavonoids in the HAE showed the presence of rutin (Rf: 0.72) in both genotypes and other unidentified compounds (Figure 1). Anthracenosides, coumarins and alkaloids were not detected, likely because of the extraction methods used. For example, alkaloid extraction requires acidic or alkaline solvents. The two species have presented a similar phytochemical profile. Flavonoids, hydroxybenzoic acids, hydroxycinnamic acids and hydroxycinnamyl amides were detected in the young aerial parts of *Amaranthus* genotypes [17]. Perdesen *et al.* [18] reported phenolic amides in other varieties of the *Amaranthus* genus (*A. hypochondriacus* and *A. mantegazzianus*). Hydroacetonic extracts have shown the greatest diversity of secondary metabolites. Acetone dissolves both hydrophilic or lipophilic components and is volatile, so 80:20 acetone-water can serve as an efficient extraction solvent. 

The UV–VIS spectra of the hydroacetonic, methanolic and aqueous extracts of both *Amaranthus* species showed peaks suggesting the presence of flavonoids and betalains (Table 3). Flavonoids induced 250 to 285 nm and 320 to 385 nm peaks [19]. The peaks between 255 and 258 nm should be linked to the presence of phenolic acids [20]. *A. cruentus* showed a λ_max_ at 536.6 and 536.9 nm in HAE and ME, respectively. *A. hybridus* showed a λ_max _at 536.9 and 537.2 nm in HAE and ME, respectively. These absorption peaks can be linked to the presence of betacyanins. HAE, ME and AE of the two species did not show characteristic λ_max_ values (450 to 470 nm) [1,21] related to betaxanthins. *In vitro*, the solvent property and neutral pH may alter the stability of the betaxanthins [22]. 

**Table 3:** UV-VIS absorption peaks of *A. cruentus* and *A. hybridus.*

	*A. cruentus*		*A. hybridus*	
**Extract**	λ_max_ (nm)	Abs	λ_max_ (nm)	Abs
HAE (1 mg/mL)	218.6	2.468	256.1	1.755
	257.6	1.749	355.1	1.797
	355.7	1.794	536.9	0.352
	386.0	1.682		
	536.6	0.312		
ME (1 mg/mL)	216.5	2.765	216.8	2.608
	318.5	1.524	255.5	1.734
	382.1	1.706	380.6	1.703
	536.9	0.327	537.2	0.361
AE (10 mg/mL)	355.1	1.858	201.2	2.734
	361.5	1.819	355.4	1.727
	372.2	1.803	366.8	1.766
	674.3	0.319		

HAE: hydroacetonic extract; ME: methanolic extract; AE: Aqueous extract.

### 2.2. Quantification of Phytochemicals

#### 2.2.1. Phenolic Compounds Contents

Table 4 summarises the results of the phenolic, tannin and flavonoid contents. Phenolic compound contents of HAE, ME and AE extracts were determined using the Folin–Ciocalteu assay. Total phenolics (TP) ranged from 7.55 to 10.18 mg Gallic Acid Equivalent (GAE)/100 mg. Polyphenols are well-known for their health benefits [13,22,23]. It is observed that HAE has the highest content of phenolics. 

**Table 4:** Phenolic compounds contents.

	*A. cruentus*				*A. hybridus*		
	HAE	ME	AE		HAE	ME	AE
**TP**	10.18 ± 0.60	7.55 ± 1.18	8.40 ± 2.69		9.75 ± 1.21	8.30 ± 0.52	7.75 ± 0.26
**TT**	7.17 ± 1.26	8.83 ± 1.76	8.50 ± 0.50		4.50 ± 1.00	10.17 ± 0.76	2.83 ± 0.29
**TF **	5.83 ± 0.27	2.90 ± 0.25	0.37 ± 0.04		7.06 ± 0.39	4.33 ± 0.27	0.53 ± 0.06
**TFV**	1.20 ± 0.07	0.56 ± 0.02	0.21 ± 0.02		1.31 ± 0.11	0.39 ± 0.03	0.09 ± 0.01

TP: total phenolics expressed in mg GAE/100 mg; TT: total tannins expressed in mg TAE/100 mg; TF: total flavonoids expressed in mg QE/100 mg; TFV: total flavonols expressed in mg QE/100 mg.

**Figure 2:**
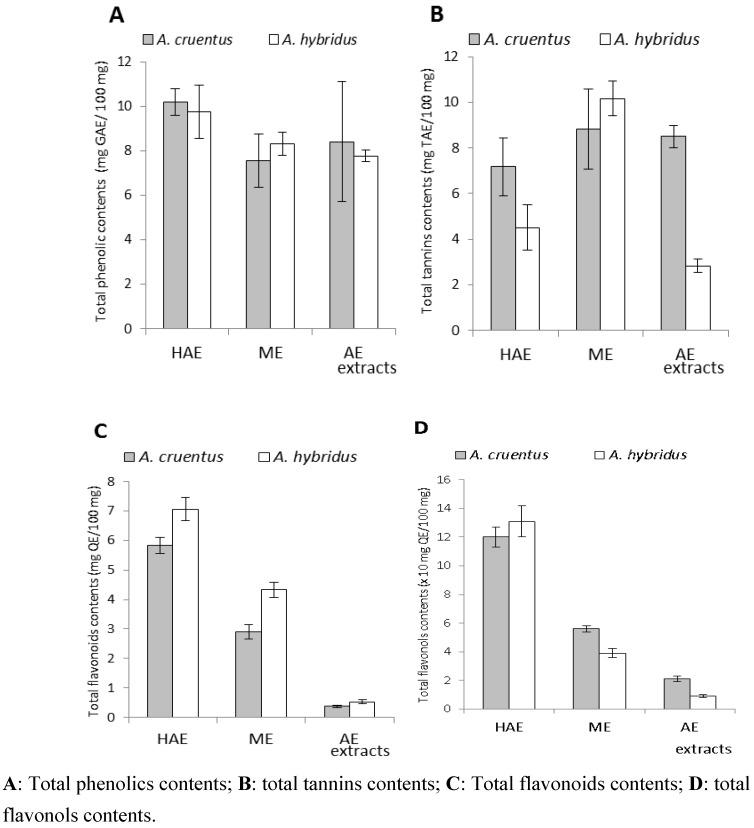
Polyphenols, tannins, flavonoids and flavonols contents.

As reported by Lamien-Meda *et al.* [24], the total phenolics in the aqueous acetone extracts were higher than those from the other solvents (Figure 2A). However, no significant difference was observed between the phenolic contents of the two plant extracts. Amin *et al.* [25] reported that the aqueous extract (boiling for 15 min) of *A. cruentus* leaves had 10.1 mg GAE/100 mg; this value is comparable to the phenolic content obtained with our aqueous extract (8.40 mg GAE/100 mg). Literature data demonstrated that the variance within the varieties of *Amaranthus* genotypes is, in general, highly influenced by environmental factors when the focus is on the content of polyphenols [26].

To specify the type of phenolic compounds, the extracted flavonoid contents were determined [27]. Total flavonoids (TF) ranged from 0.37 to 7.06 mg Quercetin Equivalent (QE)/100 mg. Flavonoid contents were also the highest in aqueous acetone extracts. Flavonoid quantities in aqueous extracts were significantly lower than those in HEA and ME extracts. It is possible that the decoction led to a loss in flavonoids in these two species. Flavonols, essential plant flavonoids, ranged from 0.09 to 1.31 mg QE/100 mg. Total flavonoid and flavonol contents ranged linearly. Data from a research report on *A. hybridus* flavonoids (rutin, isoquercetin and nicotiflirin) showed that rutin (a flavonol) was the most abundant flavonoid in the aerial parts [17].

Tannin contents in the different extracts ranged from 2.83 to 10.17 mg Tannic Acid Equivalent (TAE)/100 mg. Methanolic extracts showed the highest quantity of tannins. The fact that *A. hybridus* ME extract shows s higher amount of tannins might justify why this species is used for traditional scar drawing. The AE extract of *A. hybridus* showed the weakest tannin content (2.3 mg TAE/100 mg). These results could explain the traditional food preference for this species compared to *A. cruentus*. Taking into account the tannin content and yield of each extract, we found that *A. cruentus* and *A. hybridus* contain 1.57 mg and 0.76 mg per 100 mg of dry aerial materials, respectively. These values are significantly higher than those reported in other research [4]. Tannin drugs have anti-diarrhoeal activity [28] and permit the prevention of haemorrhages [29]. Some *Amaranthus* folk medicine uses can thus be related to their interesting phenolic, flavonoid, and tannin contents.

#### 2.2.2. Betalain Contents

In *A. cruentus* and *A. hybridus* aerial parts, the betacyanin contents were 40.42 mg and 6.35 mg Amaranthin Equivalent/100 g dry weight, respectively. The betaxanthin contents were 19.34 mg and 3.75 mg Indicaxanthin Equivalent/100 g dry weight in *A. cruentus* and *A. hybridus*, respectively. Betacyanins (red-violet pigments) and betaxanthin (yellow pigments), known as betalains (a class of secondary metabolites) [30], are water-soluble nitrogenous vacuolar pigments [31,32] present in the flowers, fruits, leaves, stem barks and roots of many Caryophyllales. Cai *et al.* [9] reported that Amaranthin and Isoamaranthin constitute common betacyanins in *A. cruentus*. The same author exhibited that the total betacyanins in the various *Amaranthus* plant materials ranged from 46 to 199 mg per 100 g of fresh plant material. The betacyanin content in fresh *A. cruentus* in the present study was 7.74 mg/100 g. These data support that the two species’ aerial parts contain low quantities of betacyanin. However, it is important to note that the betalain content depends on various parameters such as age, sunshine, and wounding. In folk medicine, betalain species are harvested when they present maximum coloration [1], which is primarily attributed to betalain pigments. The traditional uses of betalain in cancers and inflammatory and cardiovascular disease treatments have strong implications for its value.

### 2.3. Antioxidant Activities

**Figure 3:**
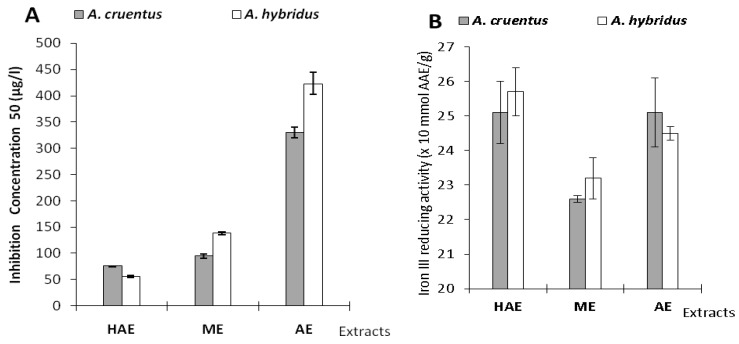
Results of *in vitro* antioxidant assays. **A**: Free radical scavenging activity represents by IC_50_. **B**: Antioxidant activity of extracts in FRAP assay.

The results of DPPH• scavenging and iron III reducing activities by the two species extracts are summarized in Table 5 and Figure 3 below.

**Table 5:** DPPH^• ^scavenging and iron III reducing values.

***Samples***	***A. cruentus***			***A. hybridus***	
	DPPH	FRAP		DPPH	FRAP
**HAE**	75.6 ± 0.5	2.51 ± 0.09		56 ± 2	2.57 ± 0.07
**ME**	95 ± 4	2.26 ± 0.01		138 ± 3	2.32 ± 0.06
**AE**	330 ± 10	2.51 ± 0.10		423 ± 21	2.45 ± 0.02

DPPH (IC_50_): IC_50_ expressed in μg ± SD/mL; FRAP: Iron (III) to Iron (II) reducing activity expressed in mmol AAE ± SD /g; HAE : hydroacetonic extract; ME : methanolic extract; AE : aqueous extract.

The radical scavenging activity by hydrogen or electron donation is a marker of antioxidant activity [33].The HAE extracts showed the highest free-radical scavenging activity for *A. cruentus* and *A. hybridus* with IC_50_ values of 75.6 ± 0.5 and 56 ± 2 μg/mL, respectively. These activities could be attributed to hydrophilic antioxidants (phenolics) and lipophilic (carotenoids) antiradical substances [15,34]. In addition, RSC (radical scavenging capacity) activity decreased in the AE extracts. Amin *et al.* [25] obtained similar results. Samarth *et al.* [35] reported that *A. cruentus* leaves’ aqueous extract (at 40 °C) had an IC_50 _value of 548 µg/mL using a DPPH assay. In our work, *A. cruentus* WE showed higher DPPH-radical scavenging activity with an IC_50_ value of 330 µg/mL. Antioxidant activities of extracts using the DPPH assay correlated well with total flavonoids. The flavonoid antioxidant properties are linked to their ability to bind metallic ions, which can be involved in free-radical genesis [19]. Radical chain reactions are broken by flavonoid hydrogen donors and also by their capacity to regenerate α-tocopherol [19]. Flavonoids possess a remarkable spectrum of biochemical and pharmacological activities that have been attributed, in part, to their antioxidant and antiradical activities [24,36].

Iron reducing (from ferric to ferrous form) activity were 2.26 ± 0.01 mmol Ascorbic Acid Equivalent (AAE)/g in *A. cruentus* ME and 2.57 ± 0.07 mmol AAE/g for *A. hybidus* HAE. *A. hybridus* HAE (2.57 mmol AAE/g) followed by *A. cruentus* HAE and AE (2.51 mmol AAE/g) showed the highest activity in the FRAP assay. Antioxidant activity measured in *A. cruentus* and *A. hybridus* HAE and ME extracts obtained using DPPH, FRAP assays showed a comparable ranking of activity as reported by Thaipong *et al.* [15]. We observed that the AE of the two species showed high iron (III) reducing power comparable to HAE activity. Antioxidant activities from the FRAP assay of the extracts were well correlated with the total phenolics. 

### 2.4. Xanthine Oxidase (XO) Inhibitory Activity

**Figure 4:**
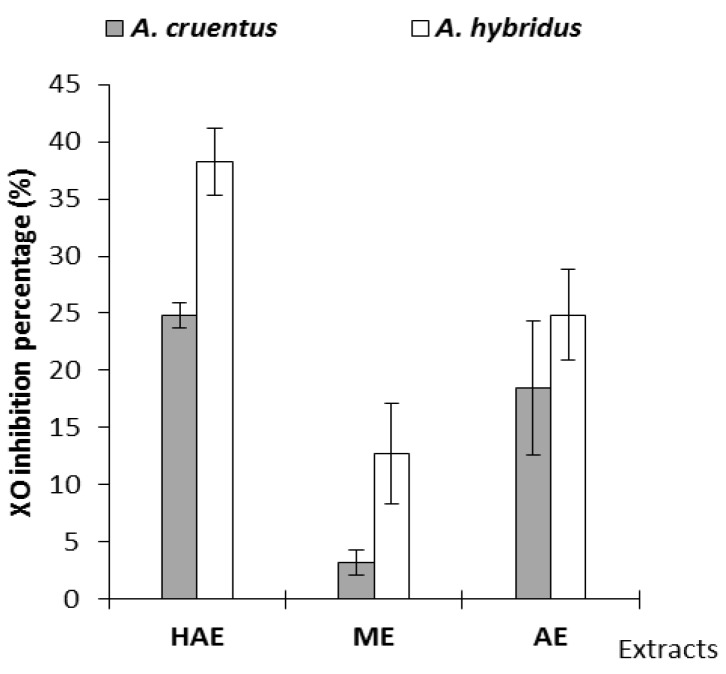
Extract xanthine oxidase (XO) inhibitory percentages.

Extracts (HAE, ME and AE) of each species were tested for XO inhibitory activity at 100 µg/mL in the assay mixture [37]. XO inhibition percentages were 3.18 ± 1.10% and 38.22 ± 2.92% for *A. cruentus* (ME) and *A. hybridus* (HAE), respectively (Figure 4 below). Methanolic extracts showed the weakest XO inhibitory activity, specifically those from *A. cruentus* (3.18%). 

*A. hybridus* showed the best activity and it may present health benefits in the prevention of uric acid accumulation. Polyphenols, flavonoids, and saponins are potent XO inhibitors [38,39]. Kaempferol and quercetin at 50 µg/mL concentrations have shown 85 and 90% XO inhibition, respectively [28]. HAE, containing the highest flavonol content (Figure 4), has shown the best XO inhibitory activity. Flavonols constitute bioactive compounds that may be responsible for XO inhibitory activities. As for the FRAP assay, XO inhibition presented a strong correlation with total phenolic contents. 

## 3. Conclusions

This study revealed that *Amaranthus hybridus* and *Amaranthus cruentus* are rich in phenolic compounds whose biological activities are well established. The study of the biological activities confirmed the phytochemical results regarding anti-radical, antioxidant, and goute-related enzyme inhibition activities. The difference in the tannin contents explained the differences in the use of the two species; *Amaranthus cruentus* being richer in tannins, is primarily used for external applications, whereas *Amaranthus hybridus* is more flavonol-rich and less rich in tannins and is most commonly used as a food. Because studies have shown that some species of *Amaranthacea* could contain anti-nutritional factors such as oxalates, it would be important to evaluate the nutritional properties of *Amaranthus hybridus* in future studies.

## 4. Experimental

### 4.1. Plant Materials

Two commercial samples (1,500 g each species) of *A. cruentus and A. hybridus* were purchased for the study in September 2009 (average temperature of 29 °C) from a garden in Ouagadougou (Burkina Faso). The genotypes were cultivated on organic fertilized soils under watering conditions. The soil has a lateritic background. From young plants (one month old and 20 - 30 cm height vegetative growth stage), aerial plant parts (leaves and stems) of each species were harvested. The fresh materials were immediately flushed with water to remove sands and dusts. The plant materials were identified Pr. J. Millogo (botanist) and voucher specimens deposited at the herbarium of University of Ouagadougou. The air-dried (in laboratory conditions during 48 h) aerial parts of the plants were pulverized, and the powders packed in plastic bags were stored in the dark at laboratory’s temperature (25 °C).

### 4.2. Extractions

Fifty grams of powder material were extracted with 20% aqueous acetone (500 mL) under mechanical shaking for 48 h at room temperature. Hydroacetonic extracts (HAE) were filtered, concentrated under vacuum and freeze-dried (Telstar Cryodos 50 freeze-dryer). Methanolic extracts (ME) were obtained by extracting the plant material in 1/10 (w/v ratio) for 24 h at room temperature. Methanolic extracts were filtered and concentrated with a rotary-evaporation apparatus (Büchi 461) at approximately 40 °C. The remainder of the solvent was removed in air to dryness. Aqueous extracts (AE) of herbal materials were obtained by boiling with distilled water (1/10, w/v) for 30 min and then freeze-dried. The extract residues were weighed prior to packing in waterproof plastic flasks and stored at 4 °C until use. The different crude extract weights and yields were calculated and expressed as grams of extract residues/100 g of dried plant materials.

### 4.3. Chemical Analysis

Moisture was determined using the drying oven method as described by Odhav *et al.* [6]. Five grams of fresh aerial parts of each plant were dried in an oven at 105 °C for 3 h. To investigate the presence of secondary metabolites in the plant extracts, several methods were used. Flavonoids, tannins, saponins, coumarins, alkaloids, triterpenes and steroids (including cardenolids) were screened according the method of Ciulei [40]. Iridoids have been characterised by the method in Galvez *et al.* [27]. Betalain identification were performed on fresh plant materials (5 g) that were homogenized in a mortar and extracted with distilled water (25 mL) containing 250 mM ascorbate (water ascorbate extract: WAE) for 15 min. After centrifugation at 6000 rpm for 15 min, the supernatant was removed and stored at 4 °C. Aliquots of the supernatants were used to characterise betalains according to the colour changing method [41].

The thin-layer chromatography (TLC) of flavonoids [42] in the aqueous acetone extract was carried out on silica gel 60 F254 plates (Mercherey-Nagel) using ethyl acetate: formic acid: glacial acetic acid: water (10:1.1:1.1:2.6) as the eluent. Chromatograms were sprayed with diphenylboryloxyethylamin (1% in methanol) and polyethylene glycol 4000 (5% in ethanol) and visualised with a 365 nm lamp. Various flavonoids (rutin, quercetin, quercitrin, kaempferol, apigenin, genistein, chrysin, acacetin, luteolin and myricetin) were used as references.

UV/vis absorption spectra were collected between 200 and 700 nm with a CECIL CE 2041 UV/vis spectrophotometer.

### 4.4. Determination of Polyphenol, Flavonoid and Flavonol Contents

Total polyphenols were determined by the Folin-Ciocalteu method as described by Singleton *et al.* [43]. Aliquots (125 µL) of extracts in a methanol solution (10 mg/mL) were mixed with Folin-Ciocalteu reagent (625 µL, 0.2 N). After 5 min, Na_2_CO_3_ aqueous solution (500 µL, 75 g/L) were added and the mixture was vortexed. After 2 h incubating in the dark at room temperature, the optical densities were measured at 760 nm against a blank (0.5 mL Folin-Ciocalteu reagent + 1 mL Na_2_CO_3_) with the spectrophotometer. The experiments were carried out in triplicate. A standard calibration curve was plotted using gallic acid (0 to 200 mg/L). The results were expressed as mg of gallic acid equivalents (GAE)/100 mg of extract. 

The total flavonoids were estimated according to the Dowd method as adapted by Arvouet-Grant *et al.* [44]. A volume of AlCl_3 _methanolic solution (0.5 mL, 2%, w/v) was mixed with methanolic extract solution (0.5 mL, 0.1 mg/mL). After 10 min, the optical densities were recorded at 415 nm against a blank (mixture of 0.5 mL methanolic extract solution and 0.5 mL methanol) and compared to the quercetin calibration curve (0 to 200 mg/L). The data obtained were the means of three determinations. The amounts of flavonoids in the plant extracts were expressed as mg of quercetin equivalents (QE)/100 mg of extract. 

The contents of the flavonols were determined by the Almaraz-Abarca *et al.* [45] method. Aliquots were prepared by mixing ethanolic extract solutions (750 µL, 0.1 mg/mL) and AlCl_3_ aqueous solution (750 µL, 20%, w/v). After 10 min of incubation, the optical densities were read at 425 nm against a blank (mixture of 750 µL ethanolic extract solutions and 750 µL ethanol). All determinations were carried out in triplicate. A standard calibration curve was plotted using quercetin (0 to 50 µg/mL). The results were expressed as mg of QE/100 mg of extract. 

### 4.5. Determination of Tannin Contents

Total tannin contents were determined by the European Community reference method [46] using tannic acid as the standard. In a test tube, aqueous extract (200 µL), distilled water (1 mL), ammonium ferric citrate (200 µL, 3.5 g/L) and ammoniac (200 µL, 20%) were mixed. After 10 min, the optical densities of the samples were measured at 525 nm against a blank (200 µL aqueous extract + 1.2 mL distilled water). The data are the mean of three determinations. The results were expressed as mg of tannic acid equivalents (TAE) per 100 mg of extract (mg TAE/100 mg extract).

### 4.6. Determination of Betalain Contents

For the determination of betalain contents, the fresh materials were prepared similar to that for betalain detection. Betalains were quantified photometrically at 536 nm (betacyanins) [47] and 475 nm (betaxanthins) [48] using the literature-described molar extinction coefficients (5.66 10^4^ l.cm^-1^.mol^-1 ^for amaranthin, 4.8 10^4^ l.cm^-1 ^mol^-1 ^for indicaxanthin). All experiments were performed in triplicate. Betalain contents were calculated using the following formula:

      C = A×V× MW × DF × 100 / [ε × L × W × (1 − M)]

where C is the betalain content (g /100 g dr. wt.); V is the extract volume (l); MW is the molecular weight; DF is the dilution factor; ε is the molar extinction coefficient (726.6 g.mol^-1^ for amaranthin; 308 g.mol^-1^ for indicaxanthin; L is the path length (1 cm); W is the weight of the fresh material (g); and M is the moisture (%).

### 4.7. Antioxidant Activity Evaluation

#### 4.7.1. DPPH Radical Scavenging Assay

Radical scavenging activities of the plant extracts against stable DPPH^•^** (**2,2’-diphenyl-1-picrylhydrazyl, Fluka) were determined spectrophotometrically at 517 nm using the method of Vélazquez *et al.* [49]. Extract solutions were prepared by dissolving dry extract (10 mg) in methanol (10 mL). The samples were homogenised in an ultrasonic bath. For different concentrations, aliquots (0.5 mL) from each sample were mixed with methanolic DPPH^•^ solution (1 mL, 20 mg/ml). After 15 min in the dark at room temperature, the decrease in absorption was measured. The blank sample was composed of the same amount of methanol and DPPH^• ^solution. All experiments were performed in triplicate. Radical scavenging activities were calculated by the following formula: 

      Inhibition (%) = (1 − A/A_0_) × 100

where A_0_ is the absorption of blank sample and A is the absorption of tested extract solution. DPPH^• ^radical scavenging activity against the extracted amounts in the samples was plotted as a curve. The extract concentration allowing 50% scavenging activity (IC_50_) was determined and expressed in μg/mL.

#### 4.7.2. FRAP assay

FRAP (*Ferric Reducing Antioxidant Power*) assay was carried out as described by Hinneburg *et al.* [50]. A volume of aqueous extract solution (0.5 mL, 0.1 mg/mL) was mixed with phosphate buffer (1.25 mL, 0.2 M, pH 6.6) and aqueous potassium hexacyanoferrate (1.25 mL, K_3_Fe(CN)_6_, 1%). After 30 min incubation at 50 °C, 10% trichloroacetic acid (1.25 mL) was added. The mixture was centrifuged at 2000 rpm for 10 min. The upper layer (0.625 mL) was mixed with 1% aqueous FeCl_3_ (0.125 mL). A blank without extract was prepared under the same conditions. The absorbance was recorded at 700 nm. All determinations were carried out in triplicate. Ascorbic acid was used as a standard and the Iron (III) reducing capacity was expressed as mmol ascorbic acid equivalents (AAE) /g of extract (dry wt.).

### 4.8. Determination of Xanthine Oxidase (XO) Inhibitory Activity

The XO activity, with xanthine as a substrate, was measured spectrophotometrically according the method of Ferraz Filha *et al.* [37] with slight modifications. Extracts were screened for XO inhibitory activity at a final concentration of 100 µg/mL. The assay mixture contained phosphate buffer (150 µL, 1/15 M, pH 7.5), extract solution (50 µL) and enzyme solution (50 µL, 0.28 U/mL in phosphate buffer). The reaction was initiated by adding substrate solution (250 mL, 0.15 mM in water). The absorbance stability at 295 nm was verified for 1 min. Enzymatic kinetics were recorded at 295 nm for 2 min. A negative control (0% XO inhibition activity) was prepared with 1% methanol solution instead of the extract solution. All experiments were performed in triplicate. XO inhibitory activity was expressed as the percentage inhibition of XO, calculated as:

      % inhibition = (1− V/V_0_) × 100

where V_0_ is the linear change (blank inclination) in absorbance per minute of negative control, and V is the linear change (test inclination) in absorbance per minute.

## Competing of Interests

The authors of this manuscript have no financial or personal relationships with other people or organizations that could inappropriately influence or bias the content of the paper.
